# Based on Molecular Profiling of Gene Expression, Palmoplantar Pustulosis and Palmoplantar Pustular Psoriasis Are Highly Related Diseases that Appear to Be Distinct from Psoriasis Vulgaris

**DOI:** 10.1371/journal.pone.0155215

**Published:** 2016-05-06

**Authors:** Robert Bissonnette, Mayte Suárez-Fariñas, Xuan Li, Kathleen M. Bonifacio, Carrie Brodmerkel, Judilyn Fuentes-Duculan, James G. Krueger

**Affiliations:** 1 Innovaderm Research, Montreal, Quebec, Canada; 2 Laboratory of Investigative Dermatology, Rockefeller University, New York, New York, United States of America; 3 Janssen Research & Development, LLC, Spring House, Pennsylvania, United States of America; INSERM-Université Paris-Sud, FRANCE

## Abstract

**Introduction:**

There is a controversy surrounding the existence of palmoplantar pustulosis (PPP) and palmoplantar pustular psoriasis (PPPP) as separate clinical entities or as variants of the same clinical entity. We used gene expression microarray to compare gene expression in PPP and PPPP.

**Methodology/Principal findings:**

Skin biopsies from subjects with PPP (3), PPPP (6), psoriasis vulgaris (10) and acral skin from normal subjects (7) were analyzed using gene expression microarray. Principal component analysis showed that PPP and PPPP were different from psoriasis vulgaris and normal acral skin. However gene expression of PPP and PPPP clustered together and could not be used to differentiate PPP from PPPP. Gene-wise comparison between PPP and PPPP found no gene to be differentially expressed at a false discovery rate lower than 0.05. Surprisingly we found a higher expression of several genes involved in neural pathways (e.g. GPRIN and ADAM23) in PPP/PPPP as compared to psoriasis vulgaris and normal acral skin. Immunohistochemistry confirmed those findings and showed a keratinocyte localization for those proteins.

**Conclusion significance:**

PPP and PPPP could not be differentiated using gene expression microarray suggesting that they are not distinct clinical entities. Increased expression of GPRIN1, and ADAM23 in keratinocytes suggests that these proteins could be new therapeutic targets for PPP/PPPP.

## Introduction

The skin of palms and soles is unique and very different from other regions of the human body. These differences include the absence of hair, sebaceous glands, the increase in eccrine glands activity and the striking increase in thickness of the stratum corneum. Not surprisingly the morphology of common skin diseases is different when the palms and soles are involved. In addition some skin diseases such pustular palmo-plantar psoriasis (PPPP) and palmo-plantar pustulosis (PPP) localize specifically to palms and soles. There is a controversy surrounding the existence of these two diseases as separate clinical entities or as variants of the same clinical entity [1–4]. PPP is usually defined as a chronic skin disease characterized by crops of sterile pustules with erythema and sometimes scaling on palms and soles whereas PPPP is usually defined as a variant of plaque psoriasis present on palms and soles with the presence of sterile pustules [1]. Sometimes the morphology is intermediate between these two descriptions and it is unclear if such patients have the coexistence of psoriasis and PPP or if they show various clinical presentations of the same disease. Gene expression in acral skin including skin of patients with PPPP or PPP has not been well studied. The present study uses gene expression microarray to compare gene expression in lesional skin of patients with PPP and PPPP to normal acral and non-acral skin and to skin from psoriasis vulgaris located outside hands and feet.

## Materials and Methods

### Subjects and skin biopsies

Four mm punch biopsies performed on palms or soles of patients with palmo-plantar pustular psoriasis (n = 6), palmo-plantar pustulosis (n = 3) or normal subjects (n = 7) were analyzed. These were obtained at baseline of a previously published study on the efficacy of ustekinumab in patients with PPP or PPPP [5]. In this study palmo-plantar pustular psoriasis was defined as active palmo-plantar disease morphology suggestive of psoriasis with at least one plaque of typical psoriasis outside the palms and soles or a history of typical plaque psoriasis outside the palms and soles. Palmo-plantar pustulosis was defined as active palmo-plantar morphology suggestive of palmo-plantar pustulosis without lesions of psoriasis outside palms and soles and without history of psoriasis. All patients gave written informed consent and the study was approved by an ethics board (IRB Services, Aurora, Canada). Washout before skin biopsies were 2 weeks for topical treatments, 4 weeks for phototherapy and oral treatments such as methotrexate, cyclosporine or acitretin and 12 weeks or 5 half-lives for biologics. Biopsies were immediately frozen with liquid nitrogen and stored at approximately -70°C.

For comparison purposes samples from 10 subjects with psoriasis vulgaris (non-acral) and 24 subjects with normal non-acral skin previously collected [6] were also analyzed.

### Gene array

RNA was extracted using the Qiagen RNeasy Fibrous Tissue Mini Kit (QIAGEN, Valencia, CA) and later hybridized to GeneChip HG U133 Plus 2.0 (Affymetrix, Santa Clara, CA). Raw data have been deposited in NCBI’s Gene Expression Omnibus and are accessible through accession number GSE 80047 [6].

### Immunohistochemistry

Frozen sections were cut and processed for immunohistochemistry using antibodies against GPRIN1 (Biorbyt LLC, San Francisco, CA, USA), or ADAM 23 (Lifespan Bioscience, Seattle, WA, USA). Biotin-labelled horse anti-mouse antibodies (Vector Laboratories, Burlingame, CA) were amplified using an avidin–biotin complex (Vector Laboratories) and developed with chromogen 3-amino-9-ethylcarbazole (Sigma-Aldrich, St-Louis, MO).

### Statistical analysis

Quality control of microarray chips was carried out using standard QC metrics and R package microarray Quality Control. Images were scrutinized for spatial artifacts using Harshlight. Expression measures were obtained using the RMA algorithm with an extra loess-normalization step. Since normal non-acral-skin chips where hybridized in a different batch, we took several steps to guarantee that batch effect was eliminated. We download psoriasis and normal skin expression data from the same series of geo omnibus, and used it in conjunction with our data to provide and estimation of the batch effect using ComBat [7]. Our data was adjusted to eliminate this effect. As there was a gender imbalance between normal acral and PPP patients, we also used the combat implementation of sva packge to estimate (and thus eliminate) the effect of the gender imbalance, considering the groups as covariates in the model.

Probe-sets with at least 5 samples, expression values larger than 4, and standard deviation (SD)>0·15 were kept for further analysis (44281 probe sets). Fold changes (FCHs) for the comparisons of interest were estimated and hypothesis testing were conducted using contrasts under the general framework for linear models in the R *limma* package. P-values from the moderated t-test were adjusted for multiple hypotheses using the Benjamini–Hochberg procedure. Hierarchical clustering was performed using Euclidean distance and a Mcquitty agglomeration scheme.

## Results

### PPP and PPPP cannot be differentiated based on gene expression

**Fig 1:**
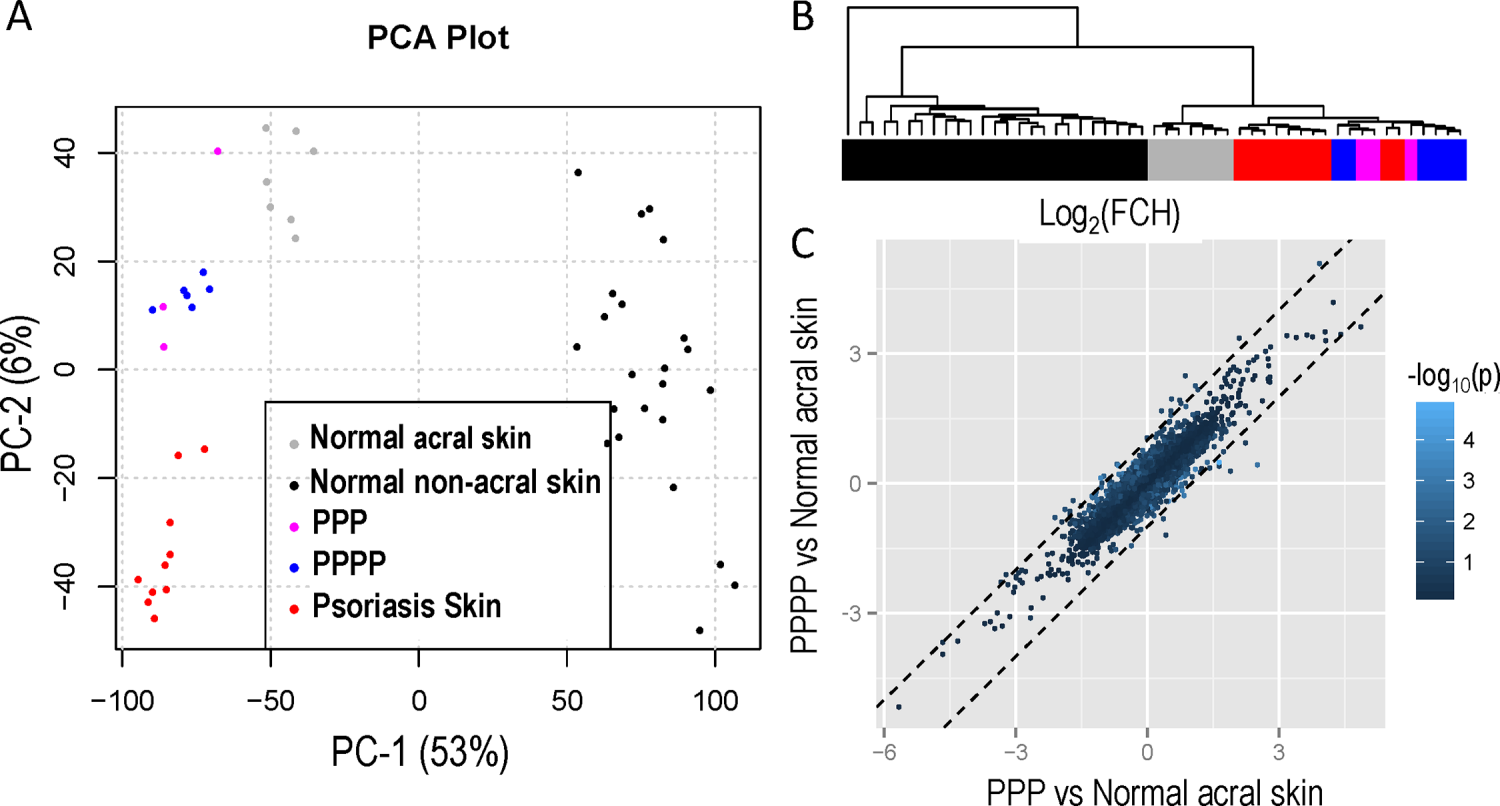
(A) Principal component analysis comparing gene expression from PPPP, PPP normal acral skin, normal non-acral skin and non-acral psoriasis vulgaris; PPP and cluster together PPPP. (B) Dendogram with unsupervised clustering of the expression profiles for all samples shows no clear distinction between PPP and PPPP as they appear in the same cluster. (C) There is a strong correlation between comparison of Fold Change (FCH) for PPP vs Normal acral skin and PPPP vs Normal acral skin.

Fig 1A compares gene expression from PPPP, PPP, normal acral skin, normal non-acral skin and non-acral psoriasis vulgaris using a principal component analysis (PCA) plot. There is distinct separation between normal acral, normal non-acral skin and psoriasis vulgaris. However gene expression in PPPP and PPP patients cluster together and cannot be differentiated. Unsupervised clustering of the expression profiles for all samples shows no clear distinction between PPP and PPPP patients (Fig 1B, dendrogram on the right y-axis) as they appear in the same cluster. Gene-wise comparison between the two groups found no gene to be differentially expressed at a FDR (False Discovery Rate) lower than 0.05.

Comparison of FCH of PPP vs Normal acral skin and PPPP vs Normal acral skin shows a strong correlation (r = 0.89) suggesting that transcriptomes of PPP and PPPP share most of their features (Fig 1C).

To study the association between the differences in the PPP and PPPP expression versus acral skin, we estimated the correlation coefficient and its distribution under the assumption of independence via simulation. We generated 1000 random samples of 3 independent variables, correspondent to PPP, PPPP and normal acral skin from a Normal distribution of mean m_*ij*_ and sd_i_ where i is the number of probe sets and j = 1,2,3 representing the mean for each gene and each group. For each random triplet we calculated the correlation between PPP vs N and PPPP vs N and hence the distribution of the correlation between PPP vs N and PPP vs Normal under the independence assumption. Under such null, the correlation distribution has a mean of 0.57 with standard deviation of 0.007 (and range 0.55, 0.59), clearly showing the significance of our results.

### Difference in gene expression between normal palms/soles and normal non-acral skin

**Fig 2:**
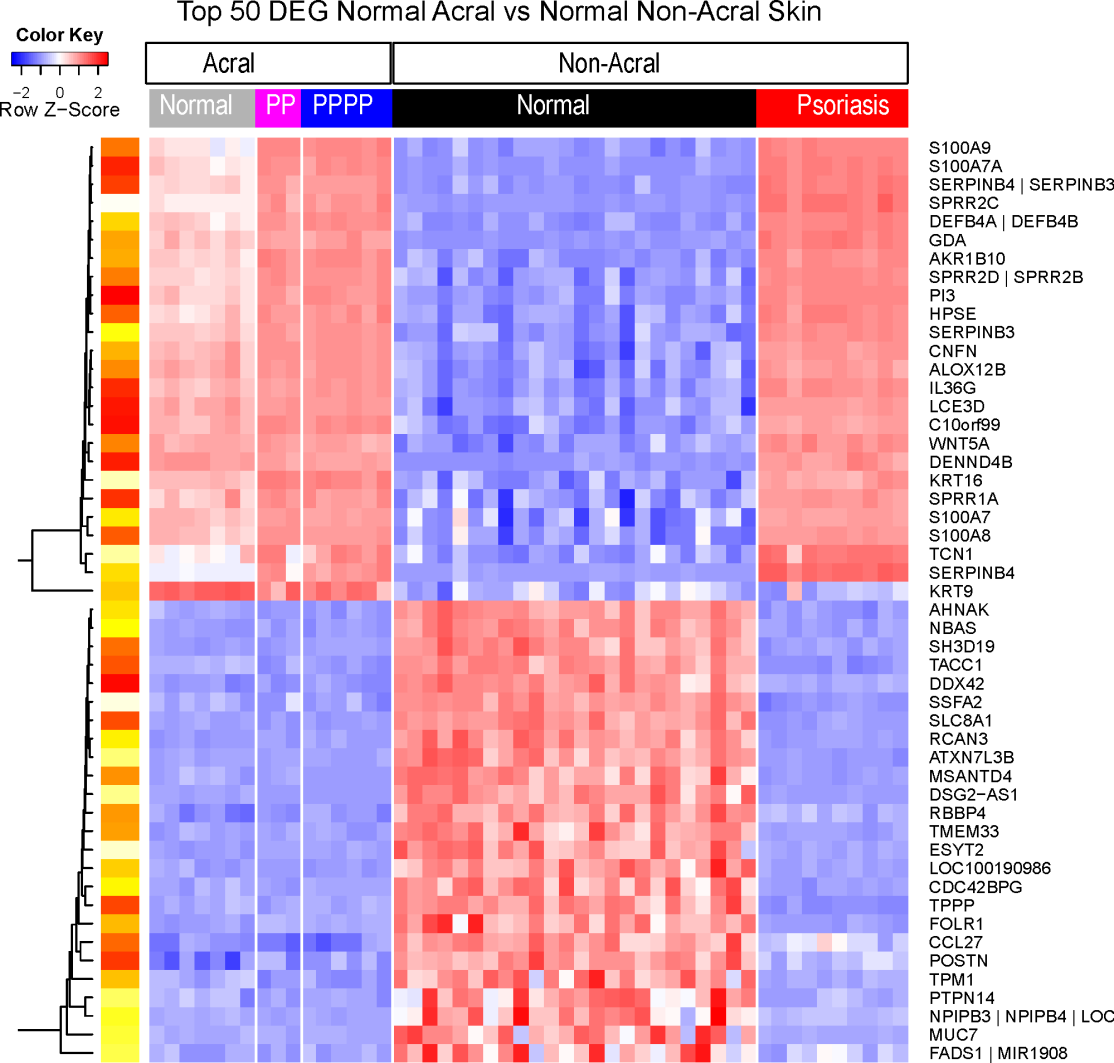
Heat map representing the expression profiles of the top 50 differentially expressed genes (DEG) of normal acral versus normal non-acral skin. Gene expression patterns from normal acral and non-acral skin are strikingly different. For DEG (FDR<0.05, FCH>2), the top 25 up and 25 down-regulated genes in terms of the fold change are presented according to an unsupervised cluster analysis. Yellow-red scale: red represents low gene expression and yellow high gene expression.

PCA plot shows striking differences in gene expression between normal palm/soles and non-acral skin (Fig 1A). A total of 4489 probe sets showed a significant difference (FDR < 0.05) of at least 2 FCH in expression between normal palm/soles and non-acral skin. Among those, there was an increase in keratin-6 (5-fold), keratin-16 (10-fold) and keratin-9 (16-fold), an increase in b-defensin4 (97 fold), lipocalin-2 (7 fold), IL-36-gamma (27 fold) and IL-36 receptor antagonist (8 fold). There was also a decrease (18 fold) in CCL27. Heat maps of representative samples confirm that gene expression in normal palms/soles and non-acral skin is different (Fig 2).

### Difference in gene expression between PPPP/PPP and normal acral skin

**Fig 3:**
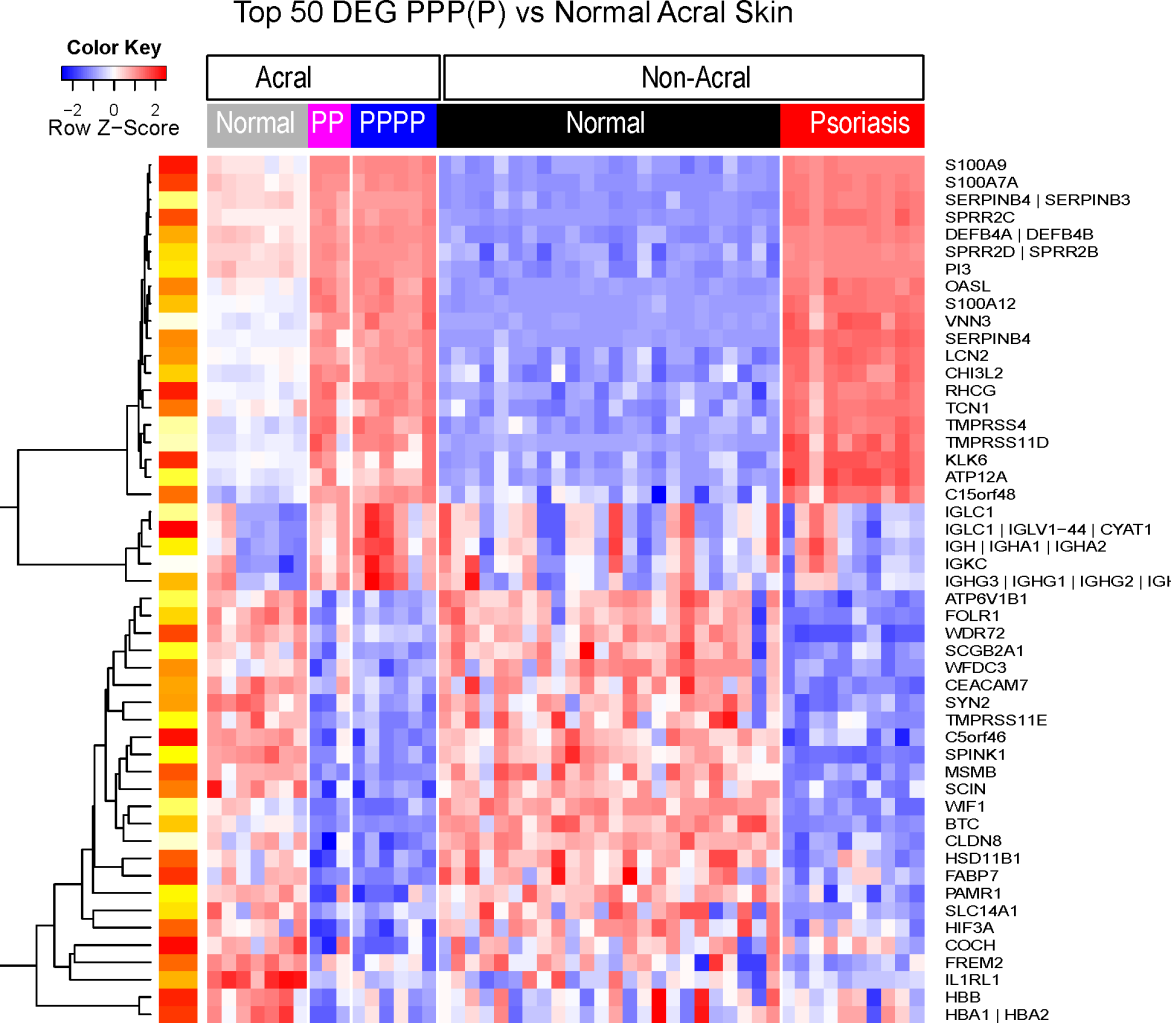
Heat map representing the expression profiles of the top 50 differentially expressed genes (DEG) of PPP/PPPP versus normal acral skin. Gene expression patterns from PPP/PPPP and normal acral skin are strikingly different. For DEG (FDR<0.05, FCH>2), the top 25 up and 25 down-regulated genes in terms of the fold change are presented according to an unsupervised cluster analysis. Yellow-red scale: red represents low gene expression and yellow high gene expression.

Important differences in gene expression between PPPP/PPP and normal acral skin can be seen on a PCA plot (Fig 1A). Since no differences were found between the two groups (PPP and PPPP), gene expression data from both groups of patients were combined and compared with normal non-acral skin. There was a significant (FDR < 0.05) difference of at least 2 FCH in gene expression for a total of 550 probe sets between PPPP/PPP and normal acral skin (the PPPP/PPP transcriptome). Among those genes there was an increase in b-defensin4 (3-fold) and lipocalin2 (3-fold), 2'-5'-oligoadenylate synthetase-like (OASL; 7 fold) and IL-36-gamma (2-fold). Heat maps of representative samples confirm that gene expression of PPPP/PPP and normal acral skin is different (Fig 3).

### Difference in gene expression between PPPP/PPP and psoriasis vulgaris

**Fig 4:**
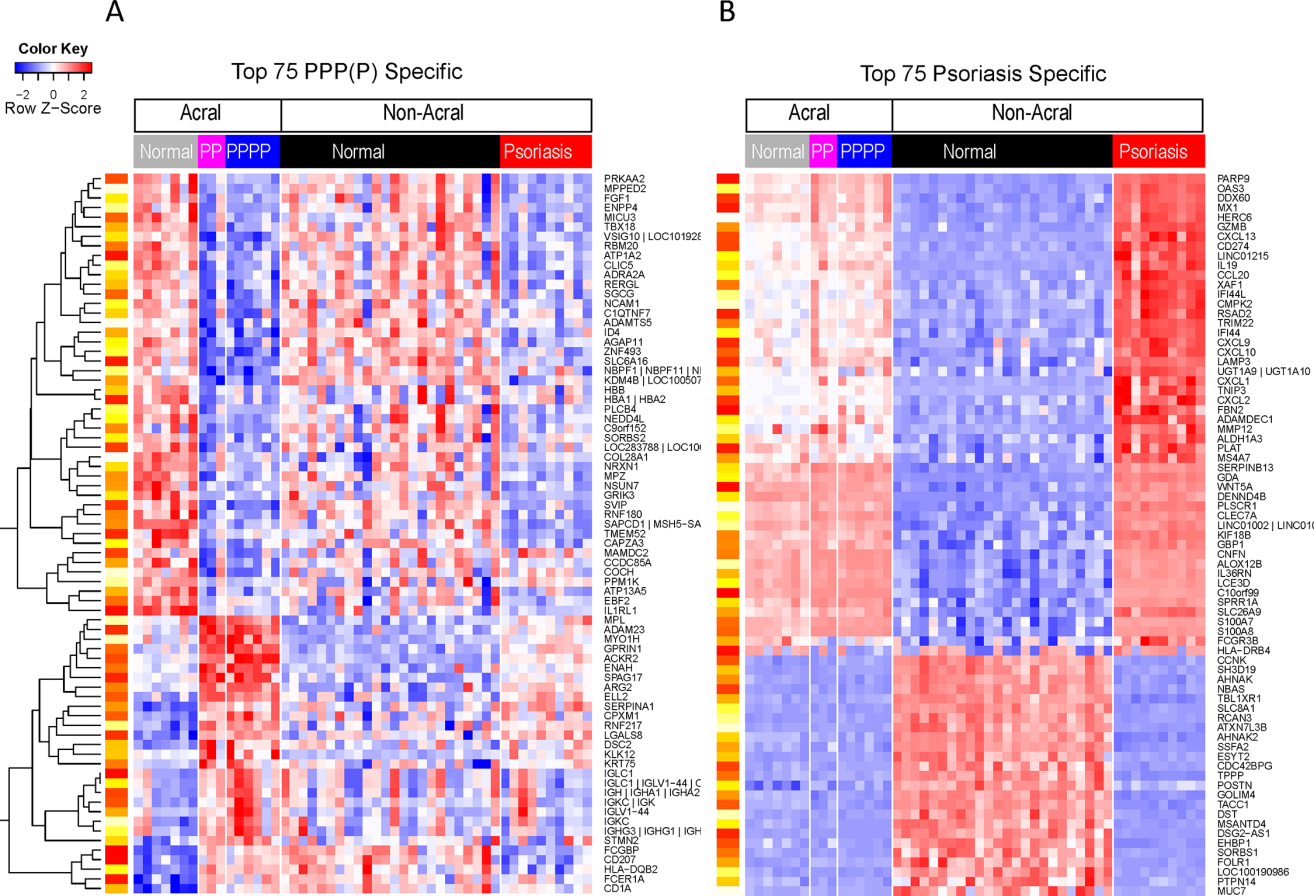
**Heat map representing the expression profiles of the top 75 PPP/PPPP specific (A) and psoriasis specific (B) genes**. The psoriasis PPP (P) specific genes are those that are differentially expressed in the PPP/PPPP versus normal acral skin and not differentially expressed in the psoriasis versus normal non-acral skin (FDR<0.05; FCH>2 in both comparisons). Likewise psoriasis specific genes are those that are differentially expressed in psoriasis as compared with normal non-acral skin but are not differentially expressed in the PPP/PPPP versus normal acral skin comparisons. In both cases the top 75 are selected based on the largest differences observed between the log2FCH of the PPP/PPPP vs acral and the psoriasis versus non-acral skin comparison. The expression of several genes, including ADAM23 and GPRIN, is higher in PPP/PPPP (lower left portion of Fig 5A) Genes are presented according to an unsupervised cluster analysis. Yellow-red scale: red represents low gene expression and yellow high gene expression.

PCA plot shows differences in terms of gene expression between PPPP/P and psoriasis vulgaris (Fig 1A). A total of 403 probe sets showed at least a 2-fold change in gene expression with a FDR of < 0.05 between PPPP/PP and psoriasis vulgaris. However, we had already highlighted the large differences between acral and non-acral skin. To account for this we compared the PPPP/PP transcriptome (DEG between PPPP/PP and normal acral skin) with those that are DEG in the psoriasis vs normal non-acral skin. Heat maps of top genes show that gene expression of PPPP/PPP and psoriasis vulgaris is different (Fig 4). One can observe that the top psoriasis genes, including IFNg genes OASL, MX1; IL17-regulated CCL20 and IL19 are not different in the PPPP/PPP as compared with normal acral skin. For genes in the PPPP/PP transcriptome however, some dysregulation is observed in psoriasis but not above the FCH cut-off.

### Increase in expression in PPPP/PPP of proteins usually expressed in neural tissues

**Fig 5:**
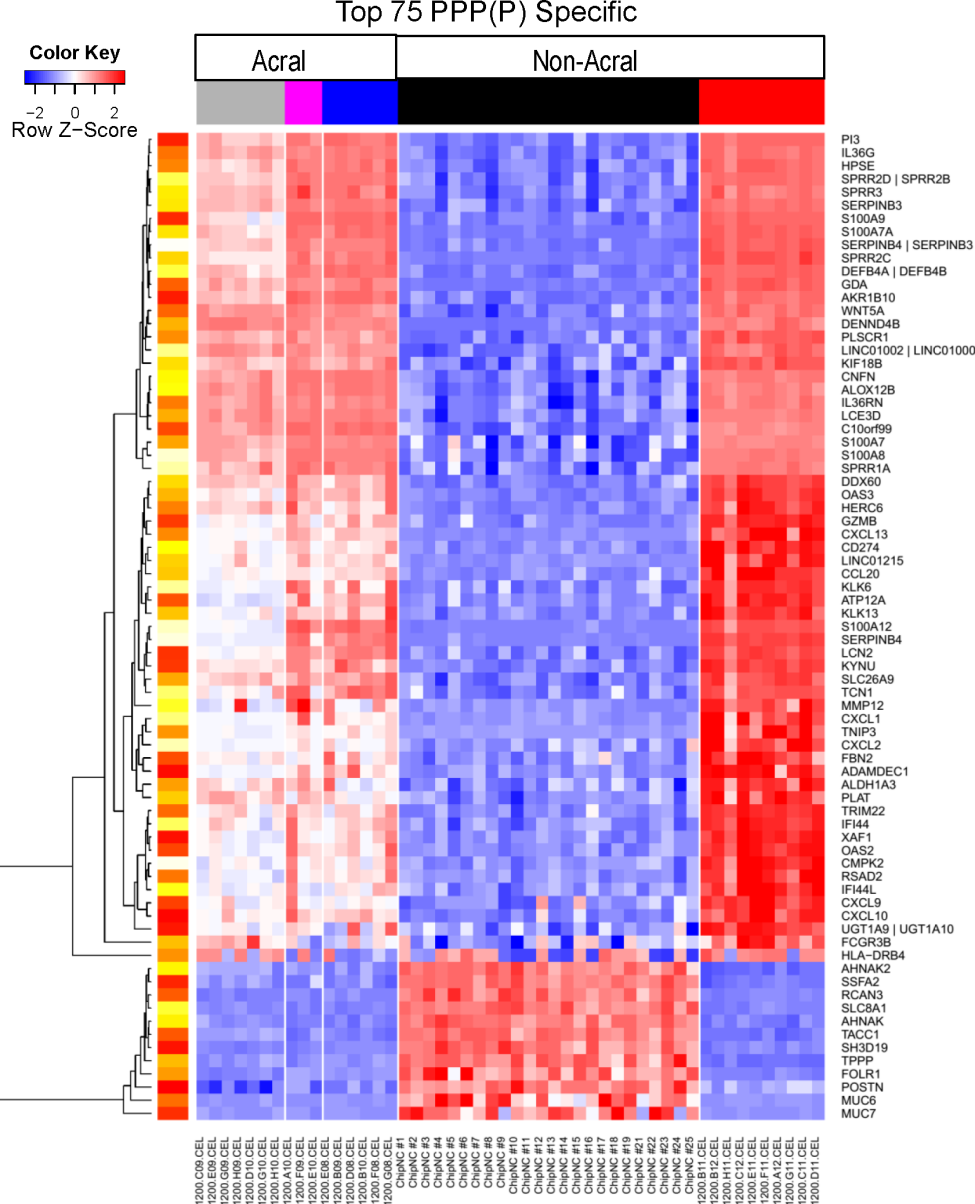
Representative images of ADAM23 and GPRIN1 localization in normal acral skin, psoriasis vulgaris and PPPP as shown by immunohistochemistry. There is an increase in expression of ADAM23 and GPRIN1 in PPPP as compared to psoriasis vulgaris and normal acral skin. There is absence of staining with a non-specific isotype antibody (negative control).

The upper right portion of Fig 4 (PPP/PPPP specific) shows genes that are more highly expressed in PPPP/PPP as compared to psoriasis vulgaris and normal acral skin. Among these are genes coding for proteins usually found in the brain and/or associated with neurons such as G Protein Regulated Inducer of Neurite Outgrowth 1 (GPRIN1), and Disintegrin and Metalloproteinase Domain-Containing Protein 23 (ADAM23). Using immunohistochemistry increased protein levels of GPRIN1 and and ADAM23 observed (Fig 5). All 3 proteins were mainly localized to keratinocytes (Fig 5). The increase in GPRIN1 and in ADAM23 expression was confirmed by RT-PCR (p<0.05 and p = 0.08 (trend with a two fold increase)) respectively; not shown).

## Discussion

To our knowledge this is one of the first study using transcriptomes to explore differences in patients with PPPP and PPP. We found that gene expression in PPPP and PPP clustered together and it was not possible to differentiate PPPP from PPP using micro-array analysis. Comparison of overall gene expression differences as well as comparisons between fold change expression of various genes did not show significant differences between PPPP and PPP. There was a strong correlation in FCH between PPP versus normal acral skin and PPPP versus normal acral skin which further supports our conclusions that transcriptomes of both conditions are not significantly different. The difficulty in differentiating PPPP from PPP at the clinical, histological and molecular level has been previously reported [2, 8, 9]. Polymorphism in the genes coding for IL-19, IL-20 and LTA (lymphotoxin alpha) have been associated with both PPP and psoriasis [8, 9]. However, polymorphism in TNF (tumor necrosis factor) promoters 238 and 308 have been associated with psoriasis vulgaris but not with PPP [10]. From a morphological point of view, there are no universally accepted criteria or descriptions to differentiate between PPP and PPPP. Patients presenting sterile pustules on an erythematous palmoplantar skin without induration, scaling and without evidence of psoriasis are often labeled as having PPP. Patients with psoriasis on the scalp, trunk and limbs sometimes having typical well demarcated, indurated and scaly plaques on their palms and soles with occasional pustules on palms and soles are usually labeled as having PPPP. Unfortunately many patients have a palmoplantar morphology that is an intermediary between these two extremes. In addition, the presence of psoriasis outside palms and soles has been reported in up to 24% of patients with a typical presentation of PPP [11, 12].

In the current study microarray analysis was also used to compare gene expression in PPP/PPPP to psoriasis vulgaris and normal acral skin in order to explore the pathophysiology of PPP/PPPP. Comparison of gene expression of normal acral skin and normal non-acral skin revealed an increase in expression of keratin 9, 6 and 16 on palms and soles which was expected given the hyperproliferative phenotype of acral skin and the relative specificity of keratin 9 for acral skin [13]. The increase in expression of antibacterial peptides b-defensin-4 and lipocalin-2 in normal acral skin as compared to normal non-acral skin is probably caused by the higher bacterial load present on acral skin. The increased in expression of b-defensin-4 and lipocalin-2 combined with the hyperproliferative phenotype of acral skin indicate that from morphological and functional point of view, the skin of palms and soles is intermediate between normal non-acral skin and psoriasis vulgaris. The decrease in CCL27, which is involved in homing T lymphocytes to the skin is intriguing and suggests that there might be mechanisms to limit adaptive immunity inflammatory reactions on palms and soles. From an evolutionary perspective intense inflammation on palms and soles could limit the ability to run, walk and use various tools, which in turn could limit survival.

Comparison of gene expression of PPP/PPPP and normal acral skin showed an increase in activation of the Th17 axis as shown by an increase in b-defensin-4 and lipocalin-2 without an increase in p40 (common subunit of IL-12 and IL-23; not shown) and an increase in activation of Th1 as shown by the increase in 2'-5' oligoadenylate synthetase (OAS)L and OAS1 expression. This confirms our previous RT-PCR findings in patients with PPP/PPPP which showed an increase in IL-17 gene expression without an increase in IL-23 [5]. Increase in IL-17 in PPP/PPPP has also been reported by others [14–16]. In addition, there was no significant difference in CXCL9 and OAS2 (not shown) which are both induced by interferon-gamma. These results suggest that the IL-12/IL-23 axis is not locally activated in PPP/PPPP. The upregulation of IL-36-gamma, IL-36-beta and of the IL-36 receptor antagonist in normal acral skin and the increase in IL-36-gamma in PPPP/PPP as compared to normal skin is interesting in view of the familial cases of pustular psoriasis reported in patients with mutations in the IL-36 receptor antagonist gene [17, 18]. Upregulation of IL-36 in acral skin may have a role for the predilection of pustular psoriasis on palms and soles. Comparison of gene expression in PPP/PPPP versus psoriasis vulgaris is more difficult to interpret as it includes differences between acral and non-acral skin and between the two inflammatory diseases. The decrease in expression of CCL20, CXCL2, CXCL10 and CXCL13 in PPPP/PPP suggests that acral keratinocytes may respond differently to cytokines such as IL-17A which regulates the production of many chemokines including CCL20. These differences also suggest a more limited inflammatory reaction on palm and soles following activation of innate or adaptive immunity to preserve the ability to walk, run and use hands for survival.

We observed an increased expression of GPRIN1 and ADAM23 at the gene and protein level in PPP/PPPP as compared to psoriasis and/or normal acral skin. The localization of these proteins to keratinocytes was unexpected. Further studies should explore the role of these proteins in PPPP/PPP as they could be new therapeutic targets. Limitations of the present study include the absence of clearly established clinical criteria to differentiate PPP and PPPP, the small number of patients biopsied. Larger studies including female-only and male-only patients are needed to confirm these findings as they will be more adequately powered to identify significant differences between PPP and PPPP subgroups.

In conclusion, transcriptome analysis of patients diagnosed clinically as having PPP and PPPP were similar but strikingly different from normal palms/soles and psoriasis vulgaris. This suggests that PPP and PPPP cannot be differentiated based on gene expression.
