# Correction: Based on Molecular Profiling of Gene Expression, Palmoplantar Pustulosis and Palmoplantar Pustular Psoriasis Are Highly Related Diseases that Appear to Be Distinct from Psoriasis Vulgaris

**DOI:** 10.1371/journal.pone.0158190

**Published:** 2016-06-21

**Authors:** Robert Bissonnette, Mayte Suárez-Fariñas, Xuan Li, Kathleen M. Bonifacio, Carrie Brodmerkel, Judilyn Fuentes-Duculan, James G. Krueger

During submission, an incorrect Fig 5 showing heat map results instead of immunohistochemistry results was erroneously inserted. Please view the correct Fig 5 here.

**Fig 5:**
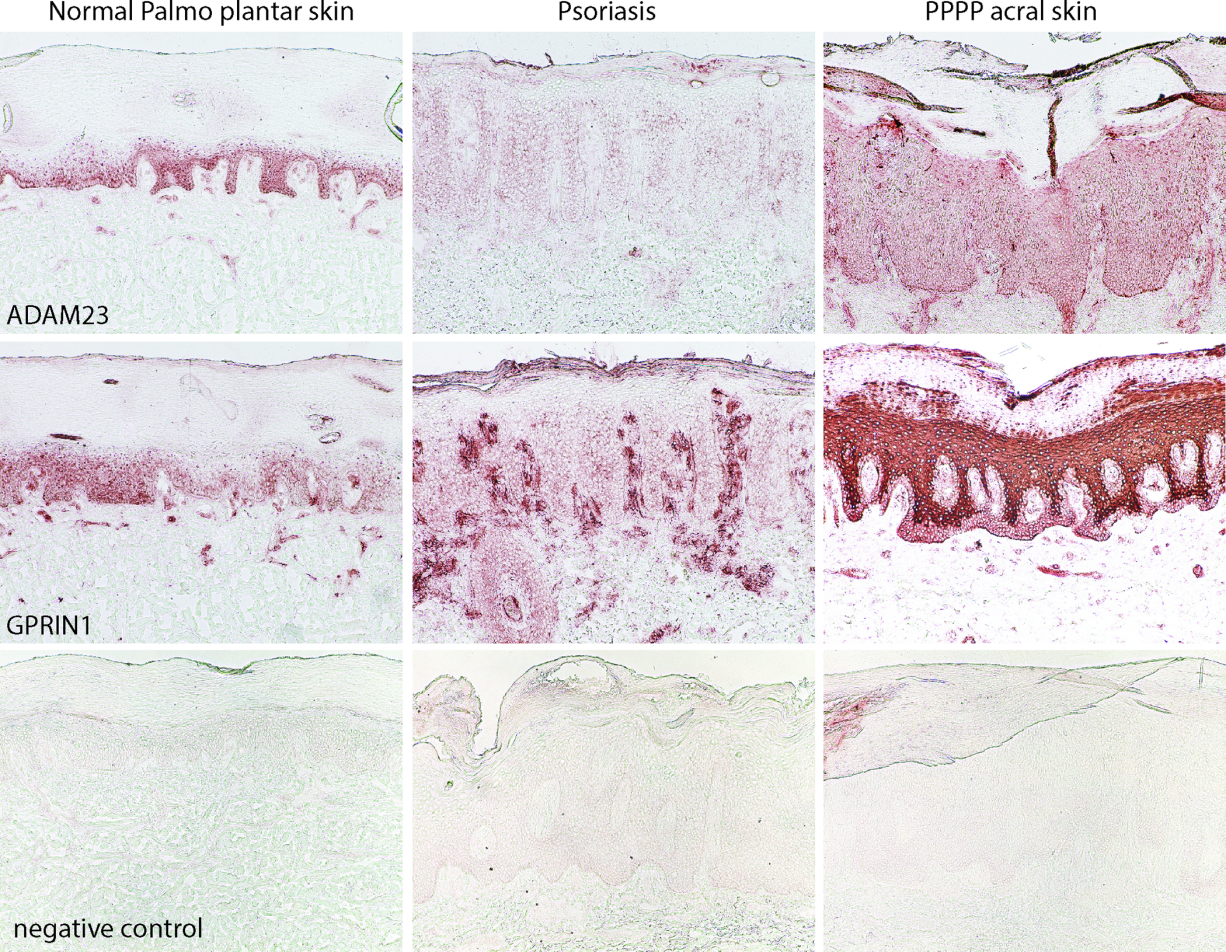
Representative images of ADAM23 and GPRIN1 localization in normal acral skin, psoriasis vulgaris and PPPP as shown by immunohistochemistry. There is an increase in expression of ADAM23 and GPRIN1 in PPPP as compared to psoriasis vulgaris and normal acral skin. There is absence of staining with a non-specific isotype antibody (negative control).
